# Supplementary figures and images for: Identification of a small molecule inhibitor that stalls splicing at an early step of spliceosome activation
Source: eLife. 2017 Mar 16;6:e23533. doi: 10.7554/eLife.23533 (PMC5354520; doi:10.7554/eLife.23533)

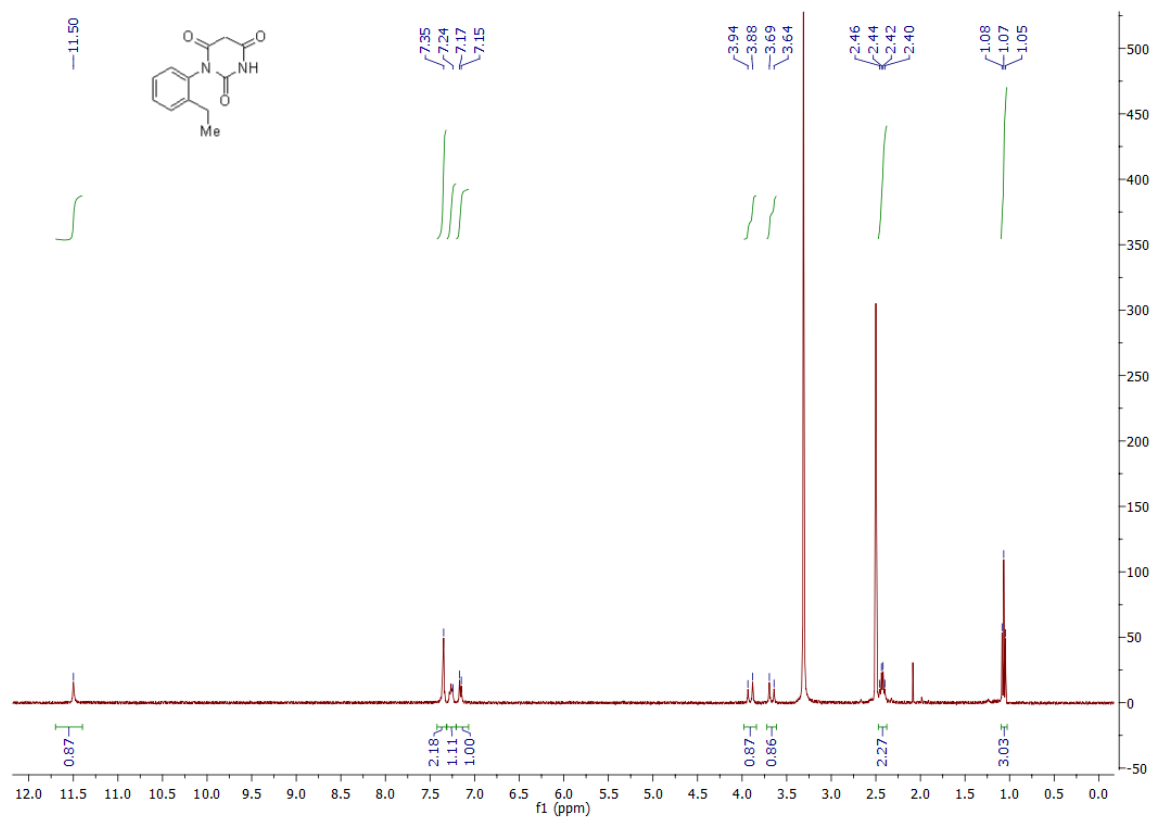

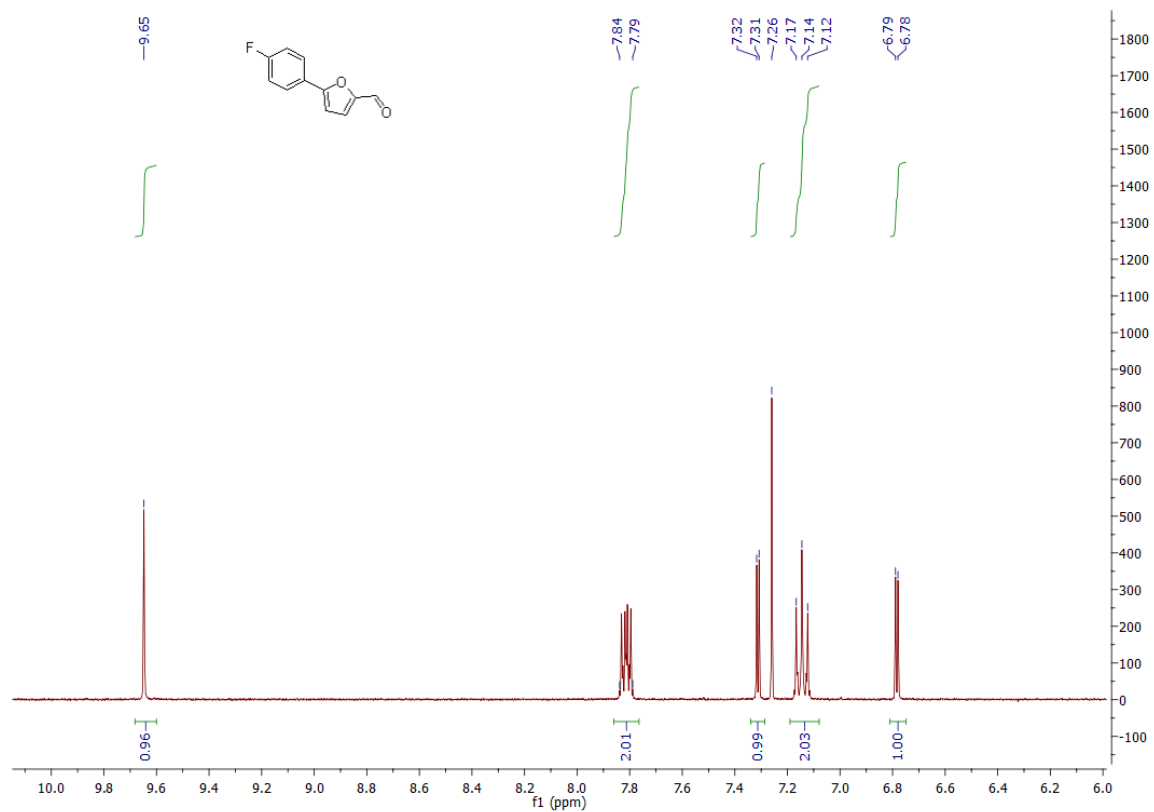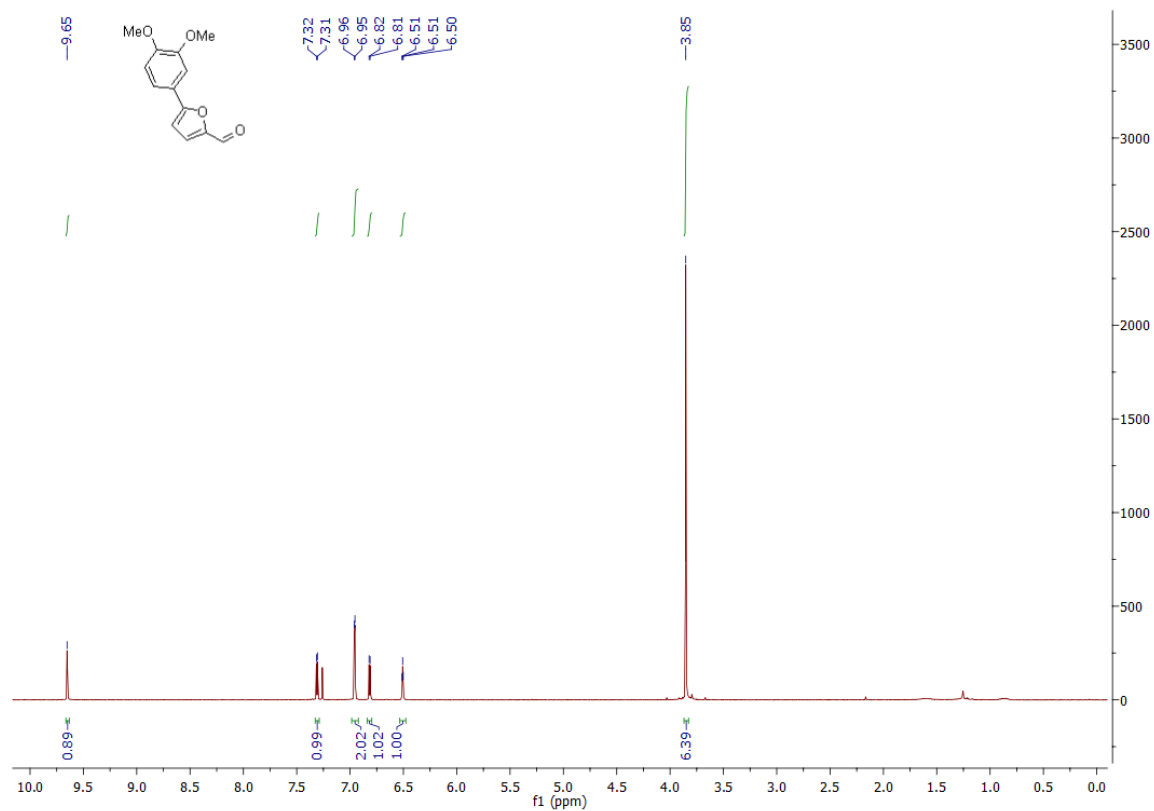

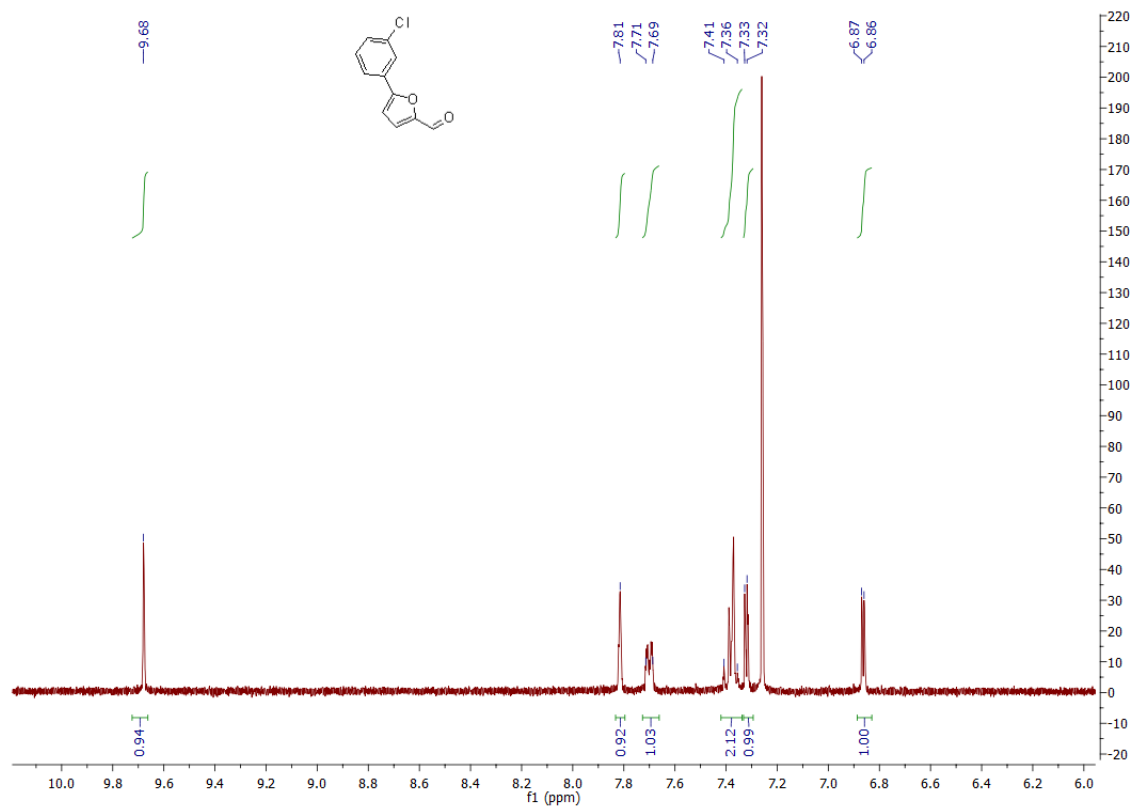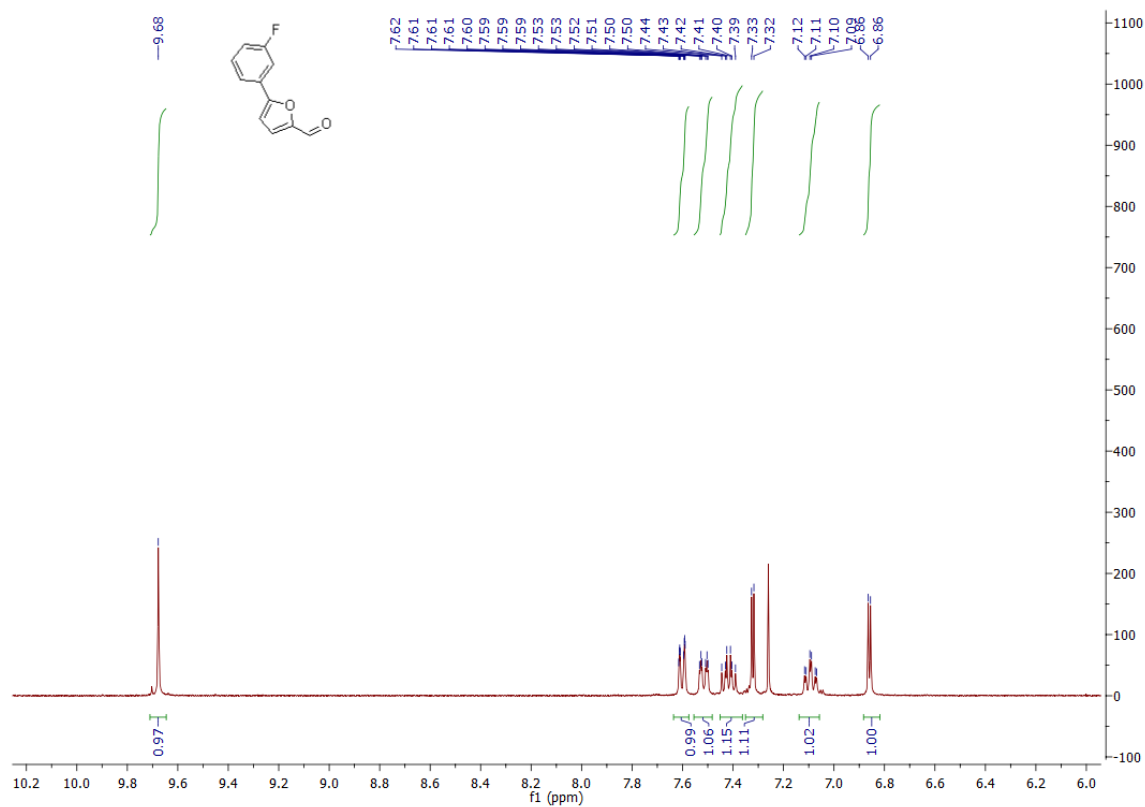

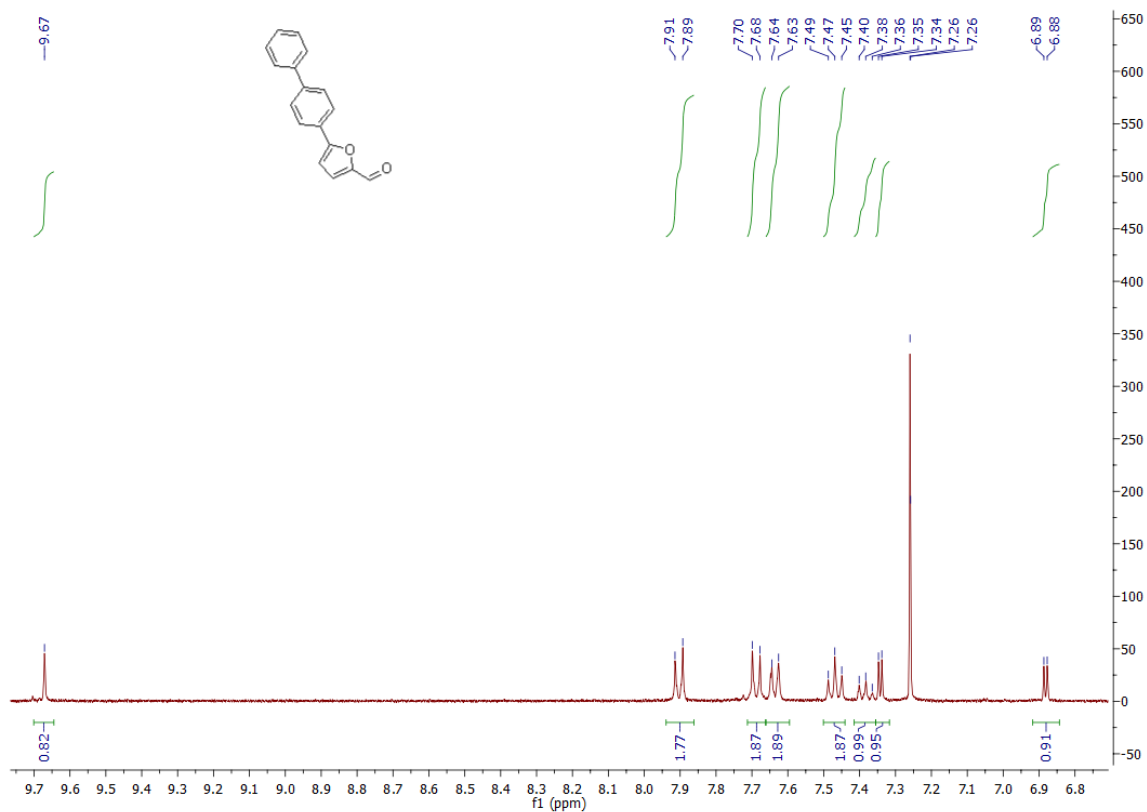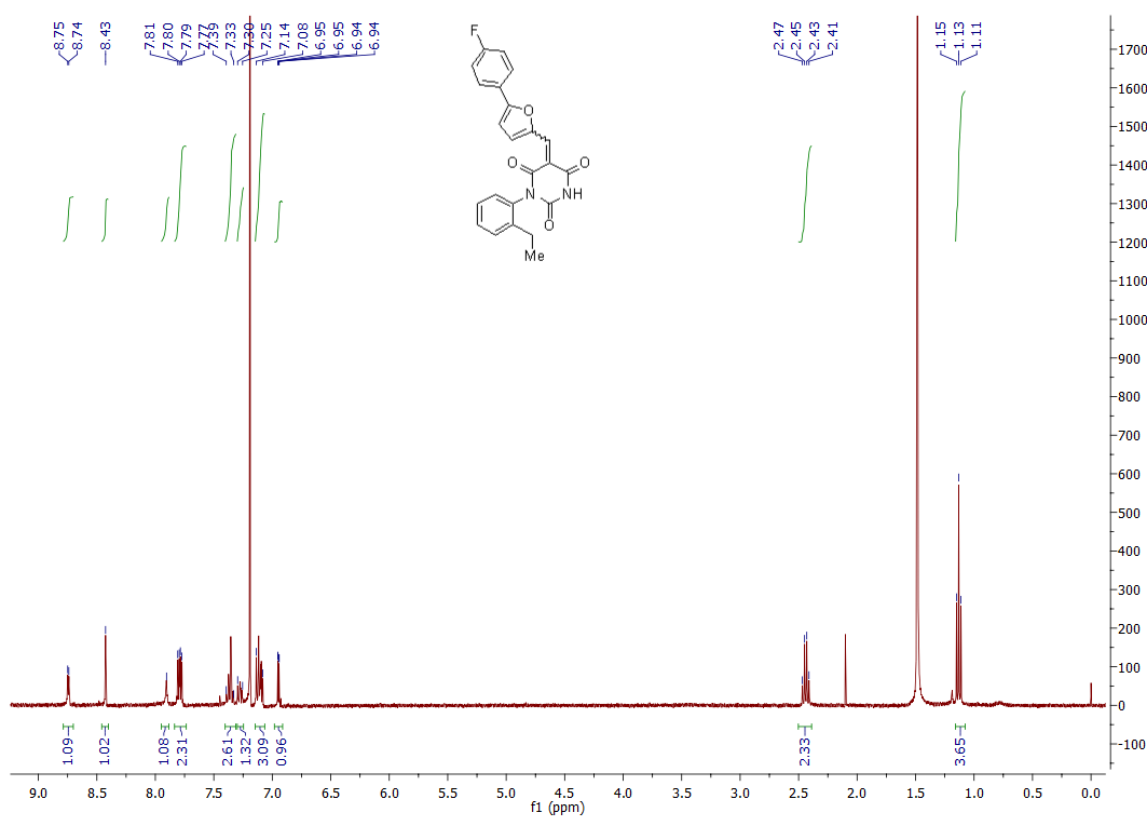

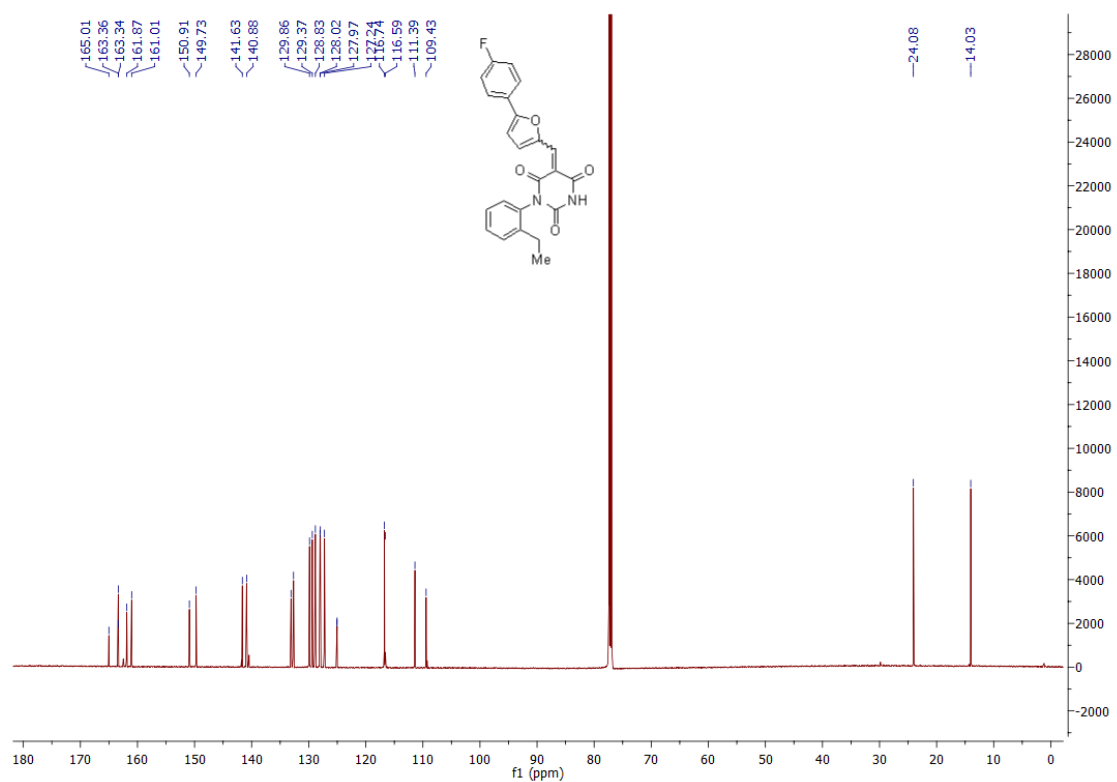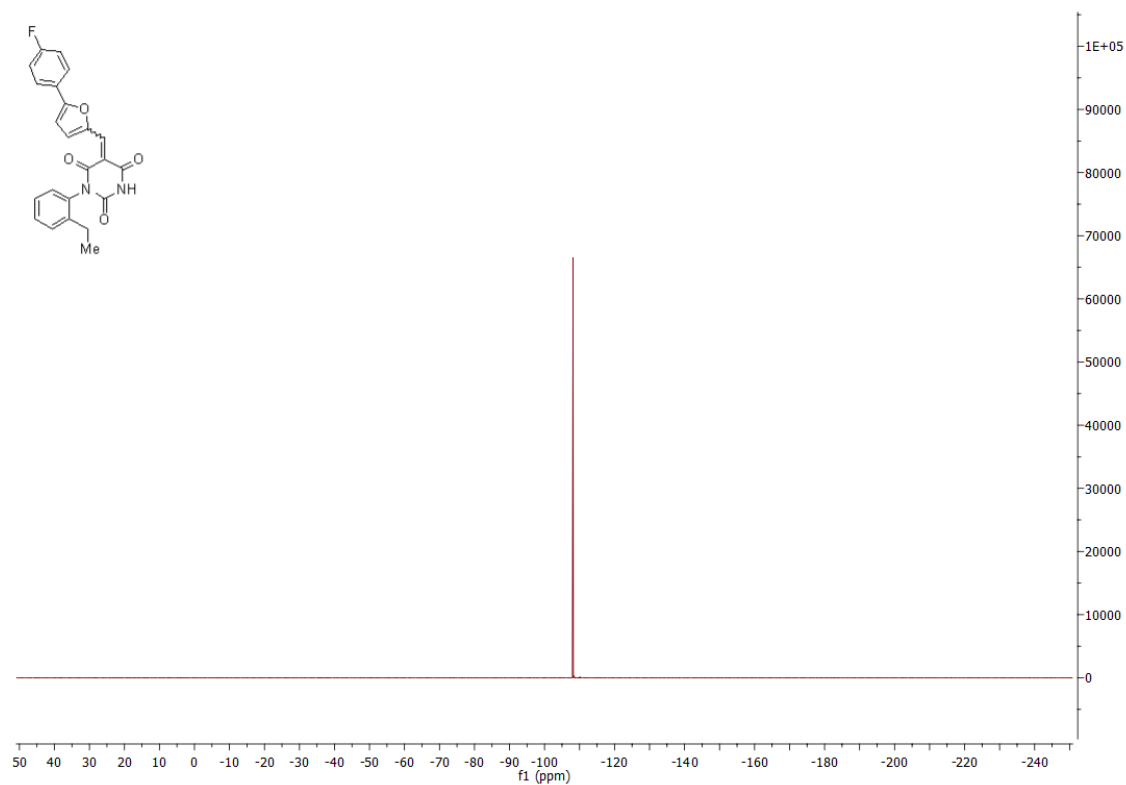

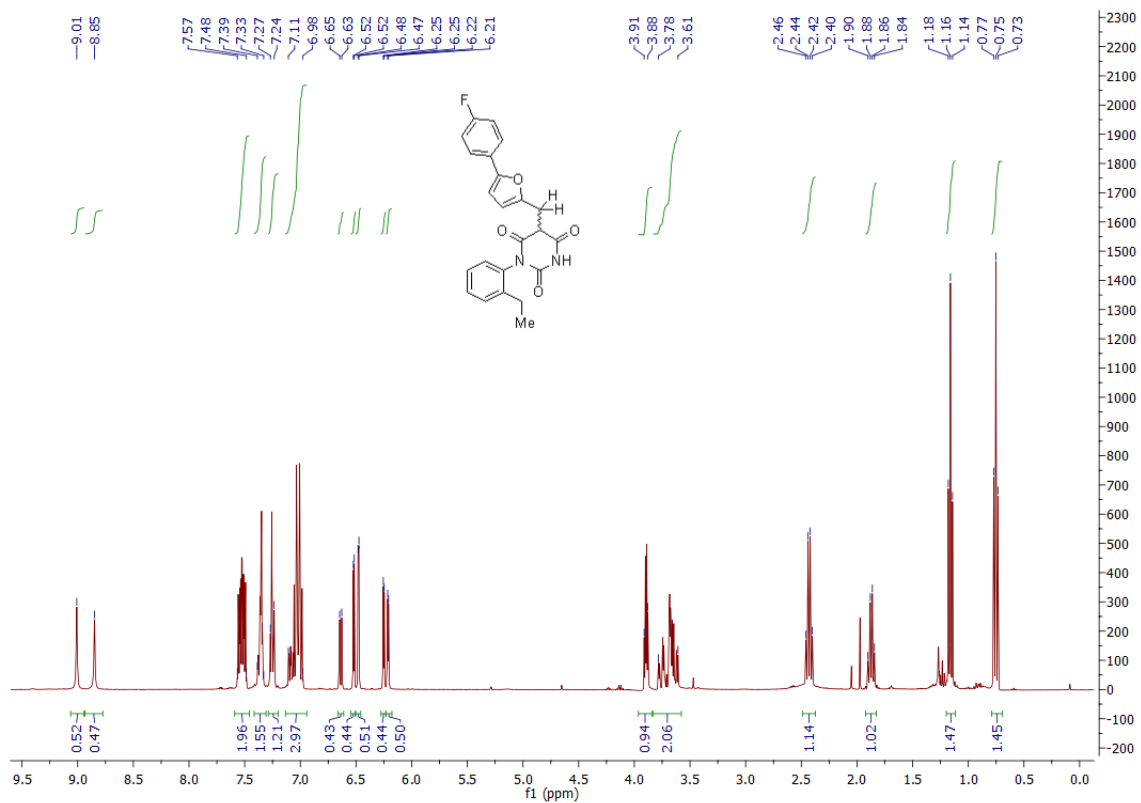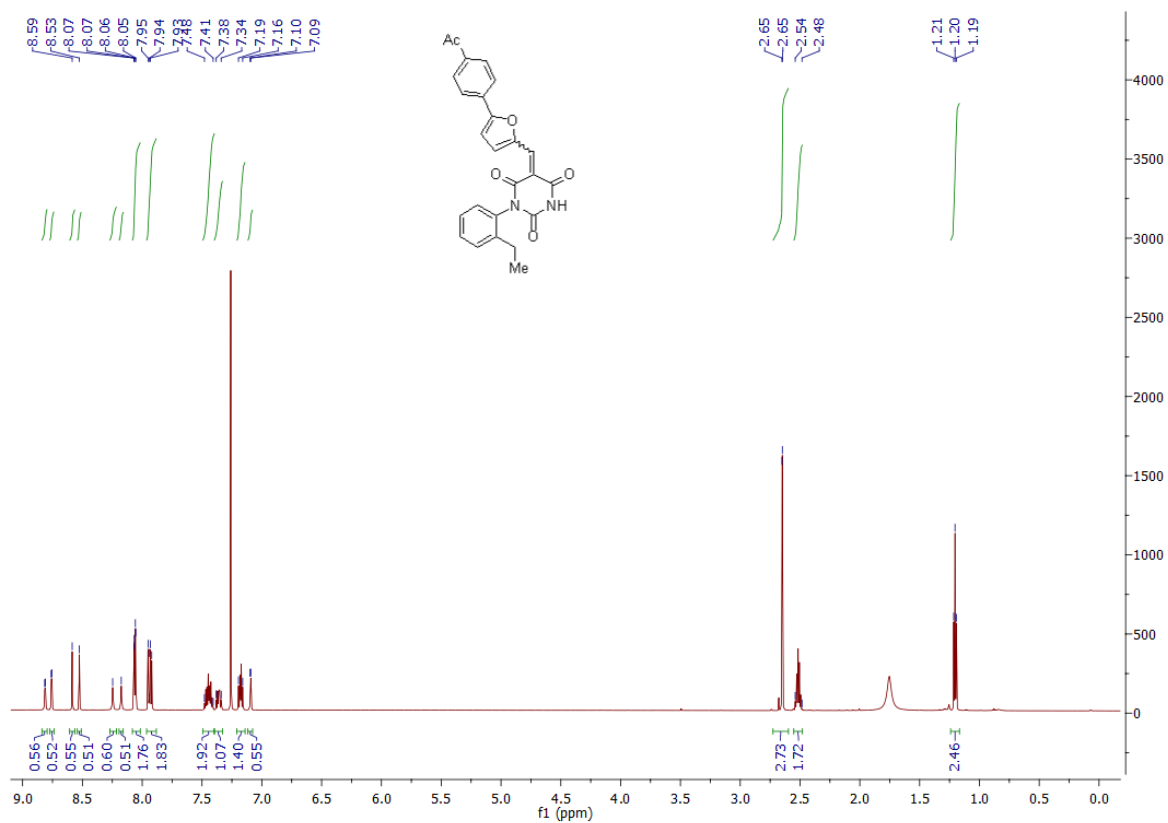

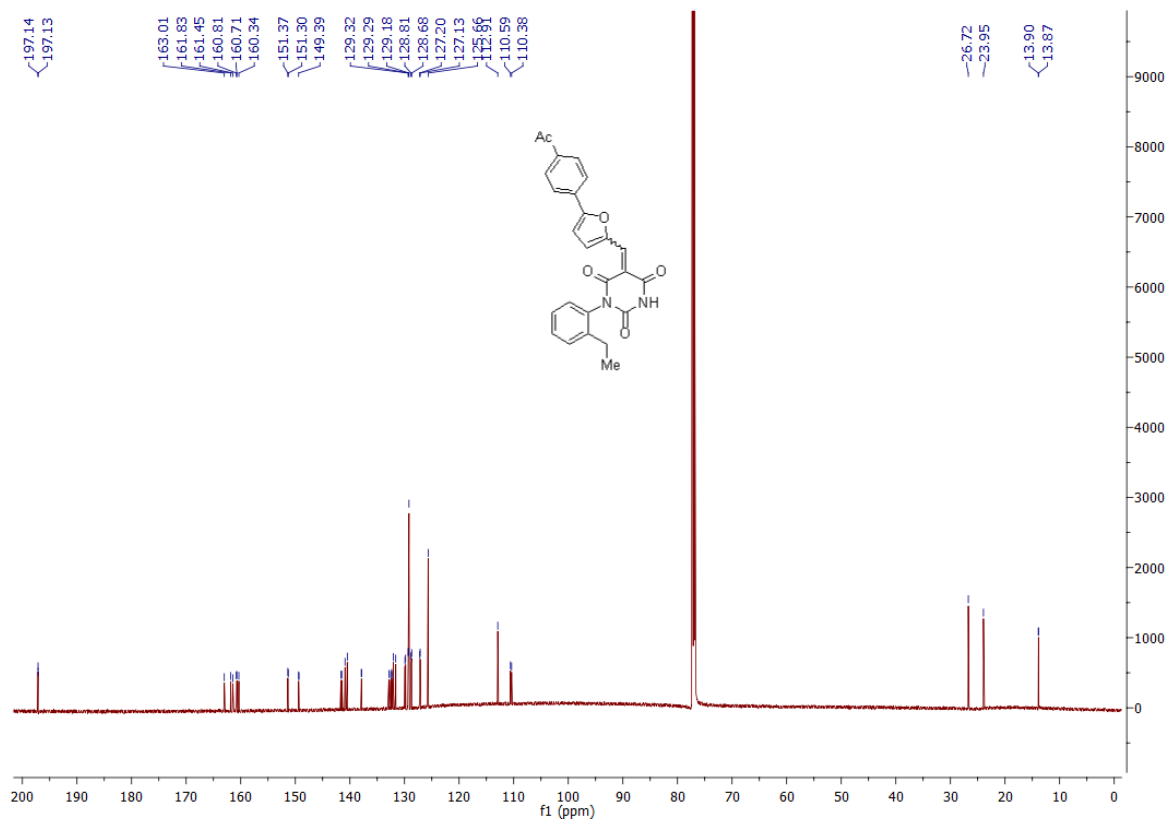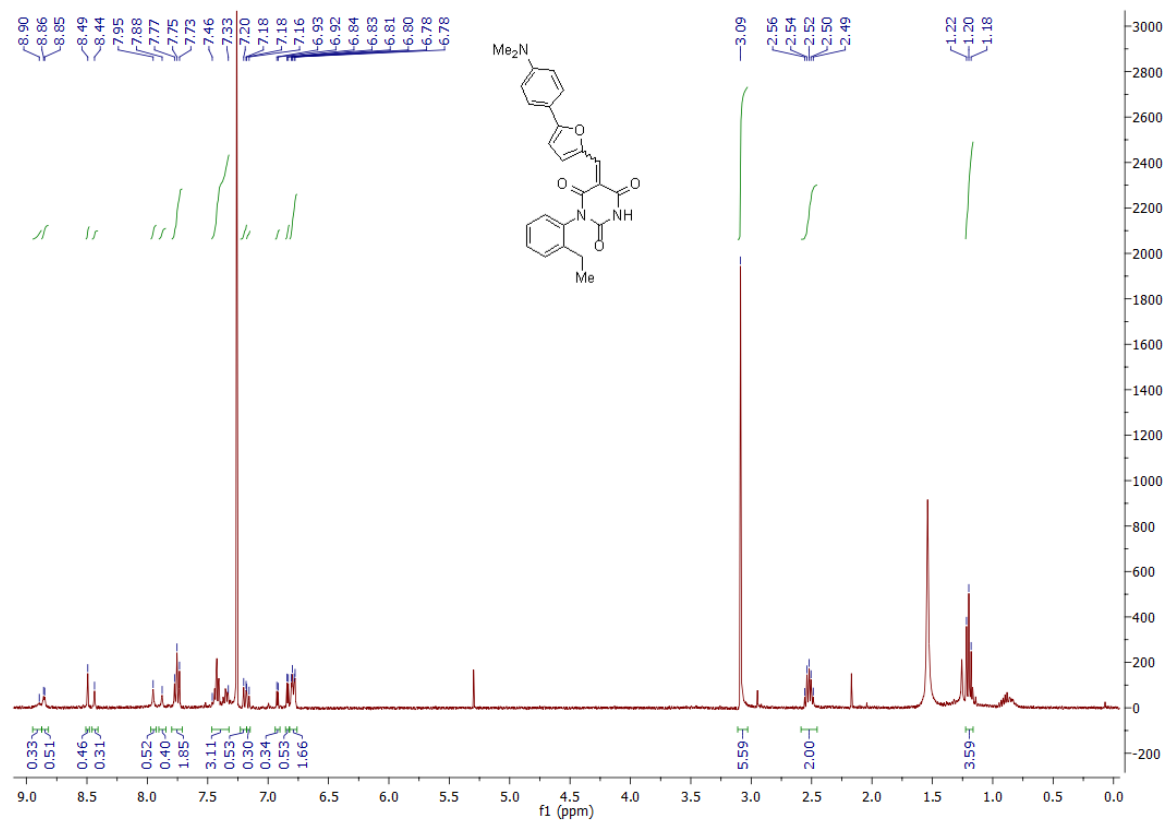

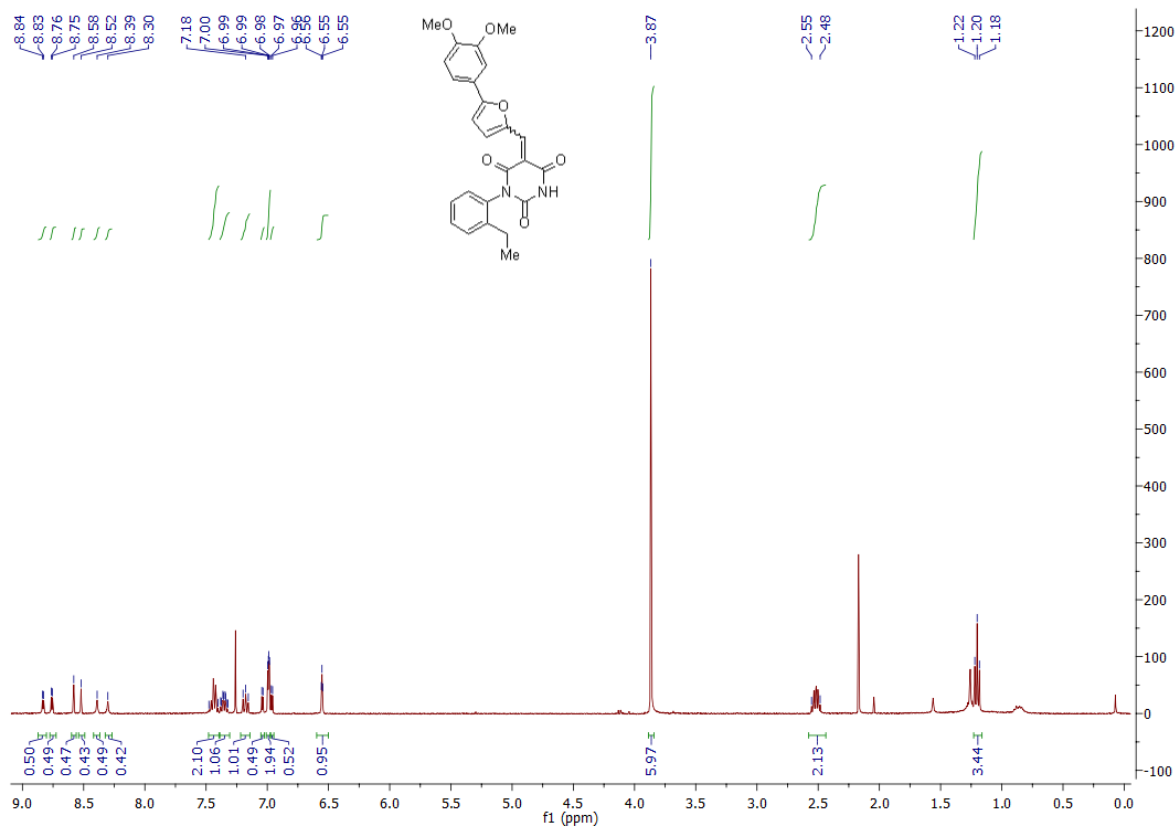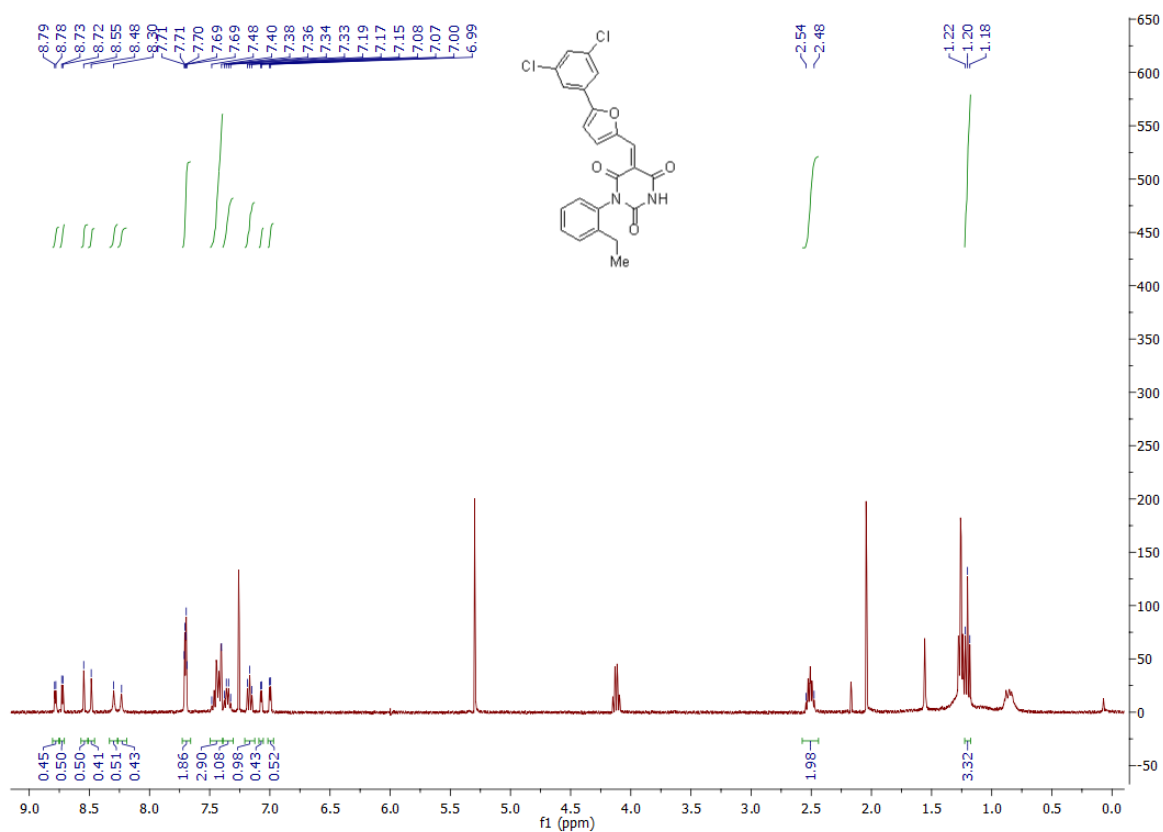

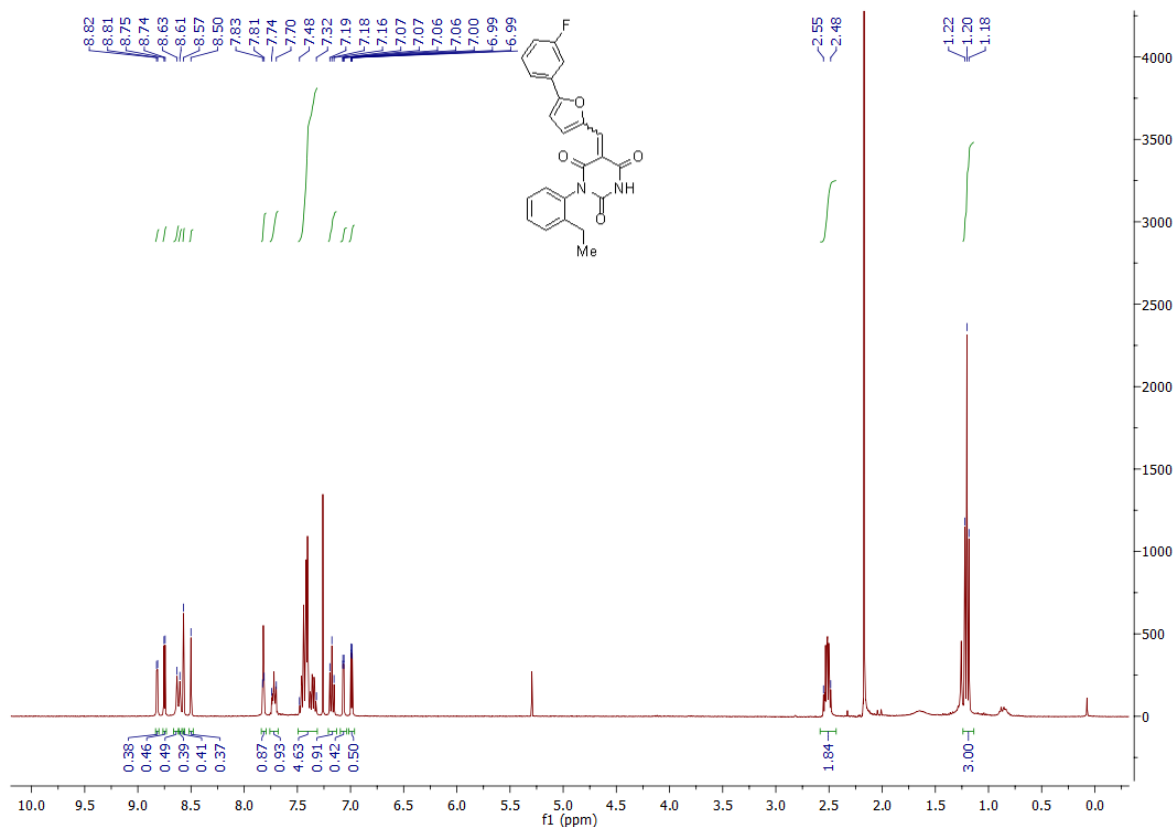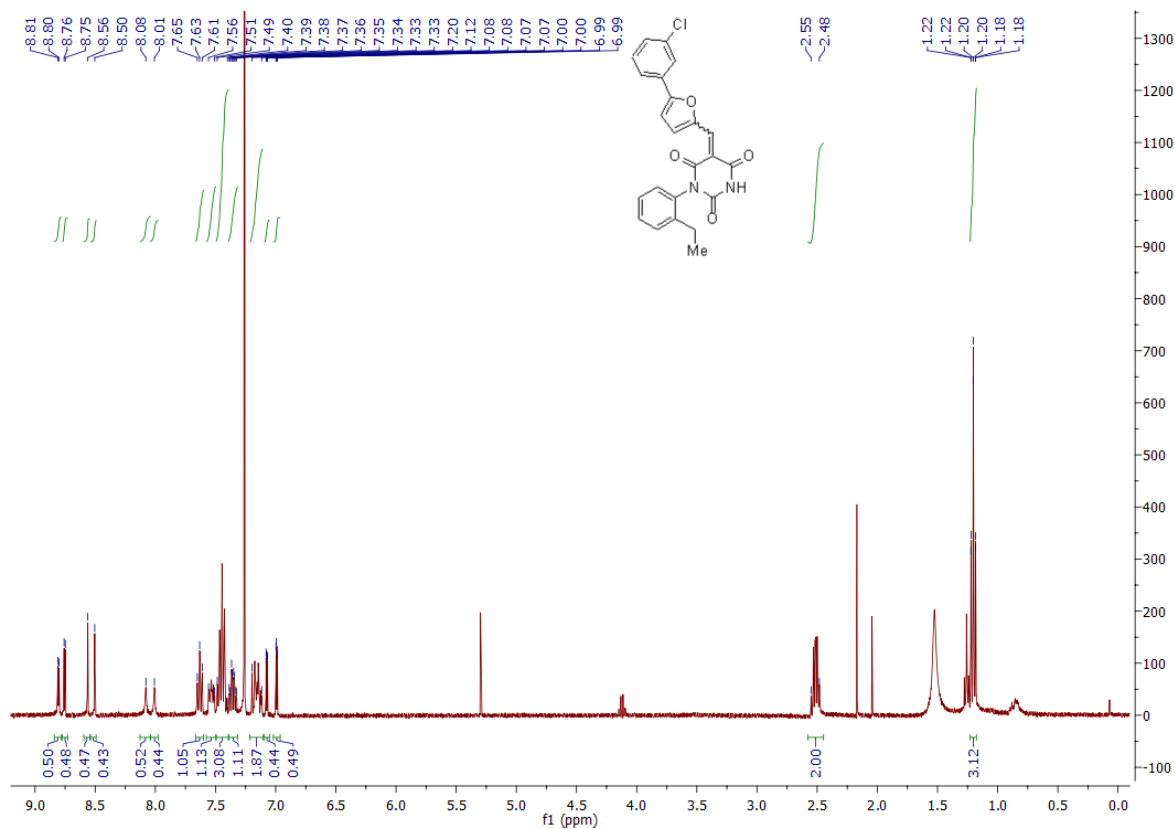

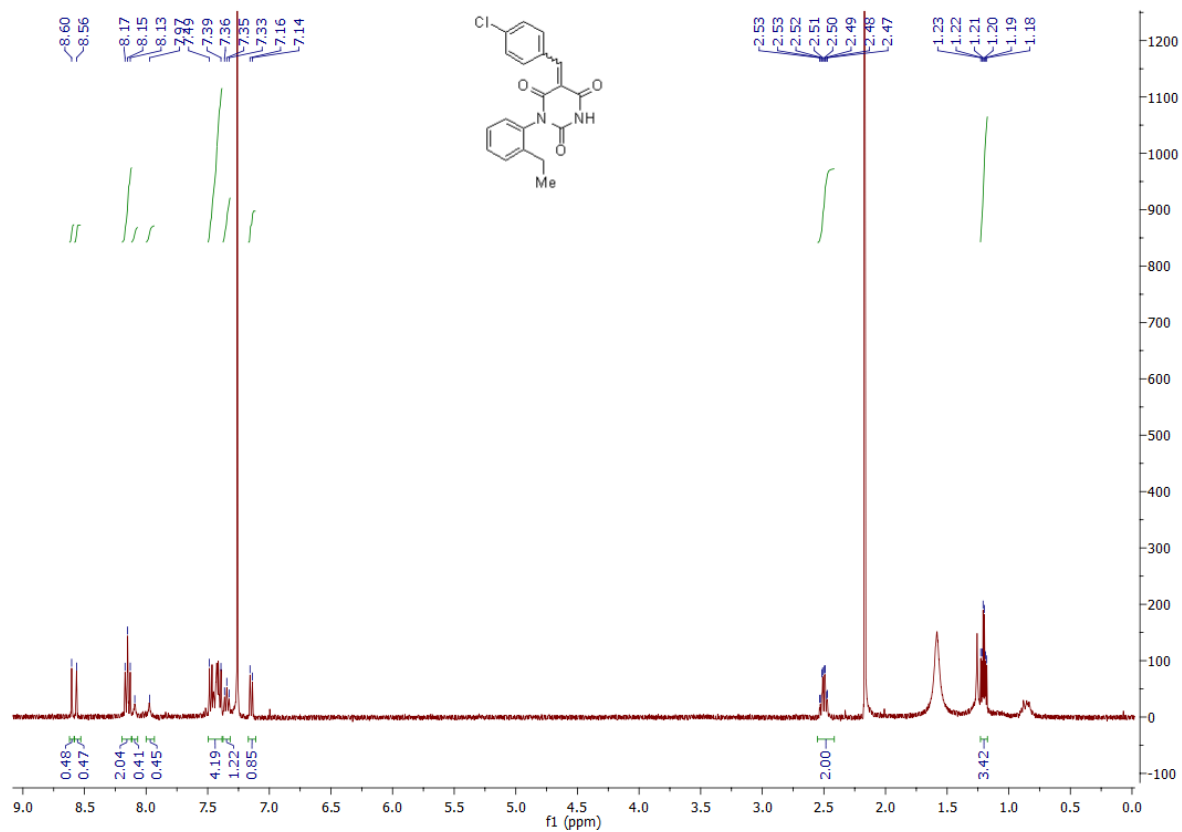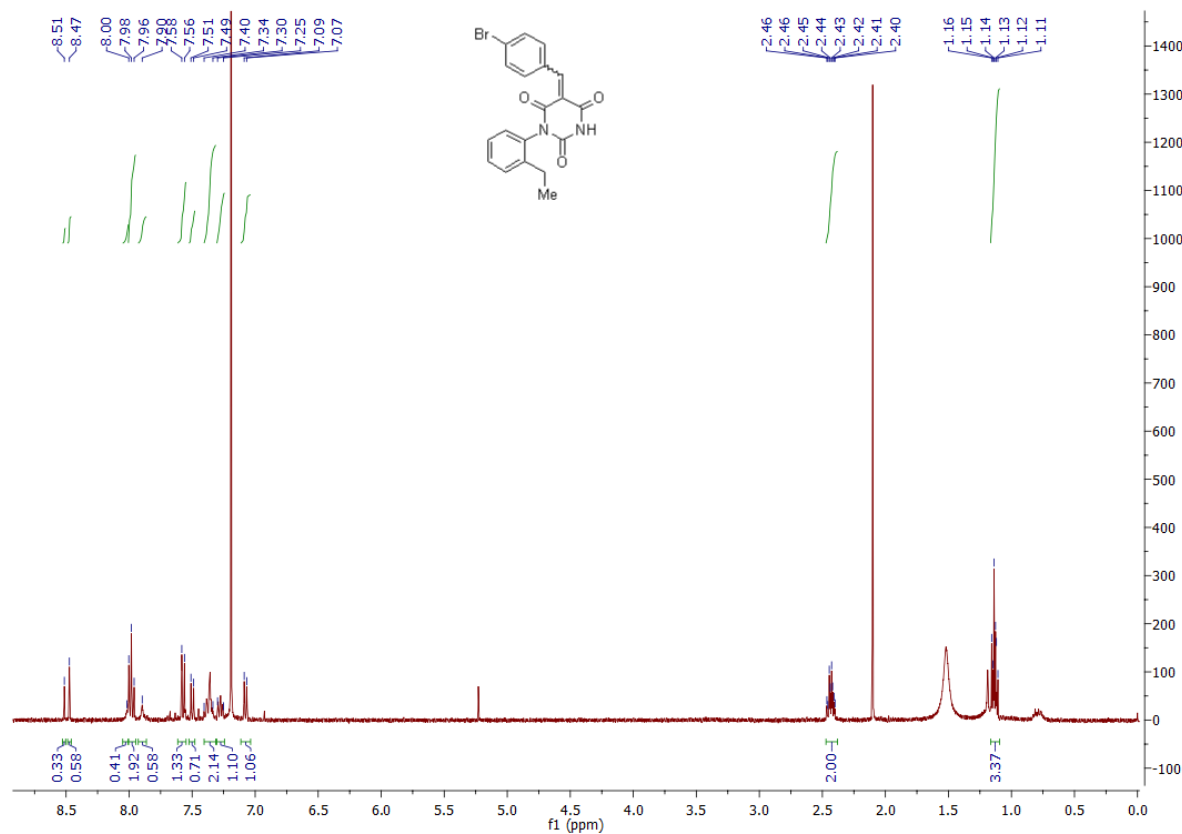

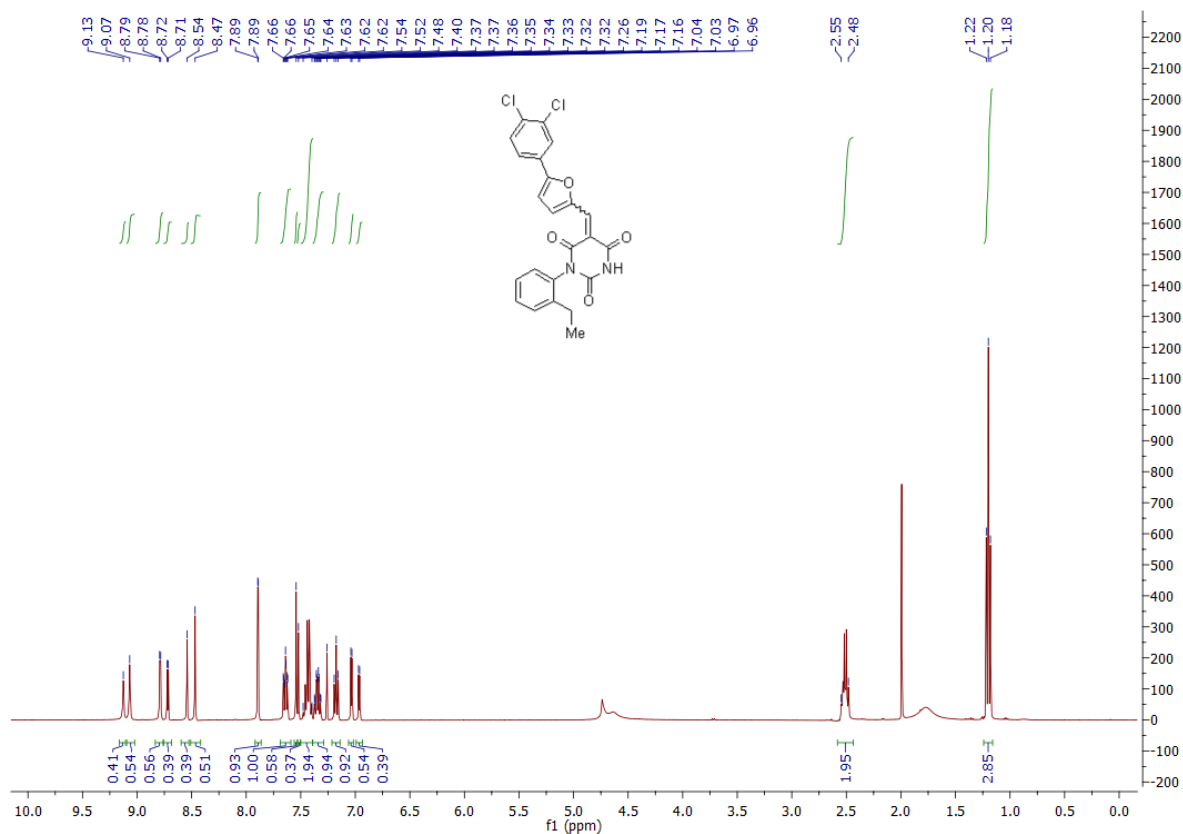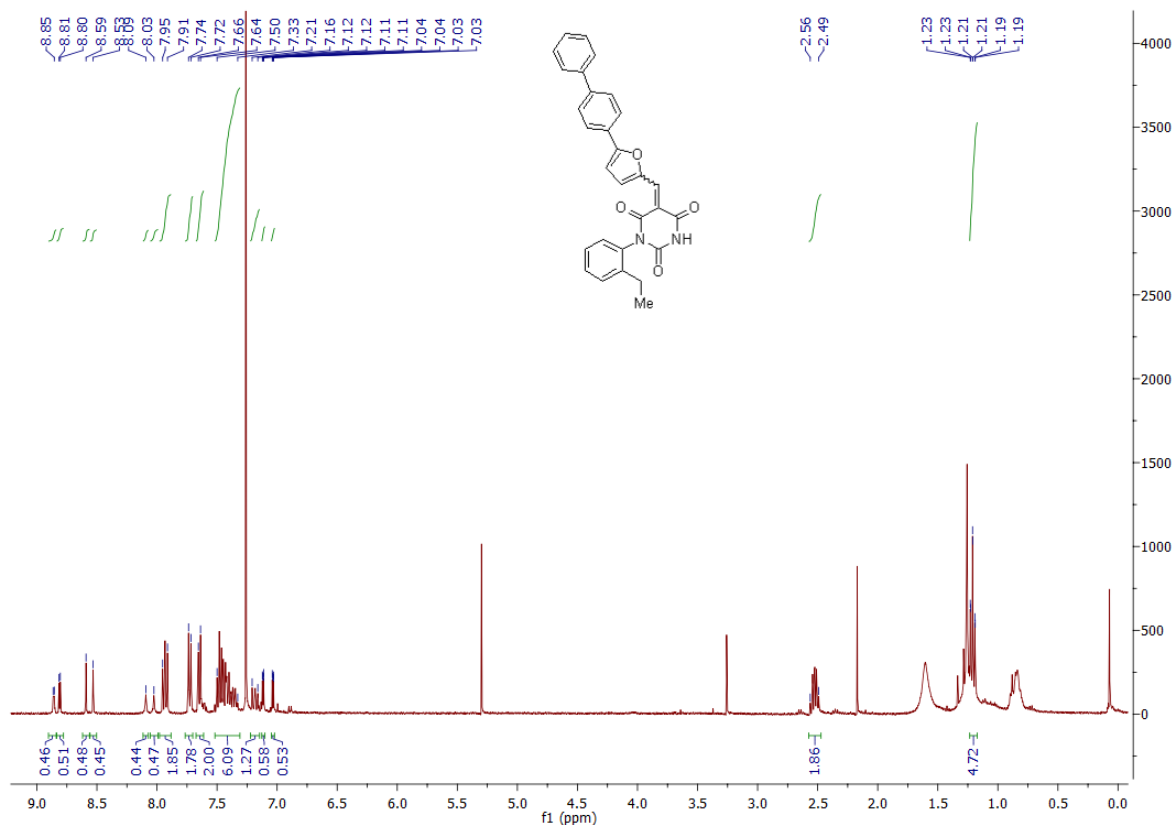

Supplement: Figure 6—source data 1. — Cp028 analogues synthesised in house and several intermediate compounds were characterised by nuclear magnetic resonance spectroscopy. A 1H spectrum is shown for all molecules. 13C and 19F spectra are provided for some of the analogues. The ppm values (in blue), the multiplicity and the 1H integrals (in green) are shown (see also description of the synthesis of each compound in Materials and Methods). DOI: http://dx.doi.org/10.7554/eLife.23533.015 [file elife-23533-fig6-data1.pdf]
