# Supplementary material for: Identification of a small molecule inhibitor that stalls splicing at an early step of spliceosome activation
Source: eLife. 2017 Mar 16;6:e23533. doi: 10.7554/eLife.23533 (PMC5354520; doi:10.7554/eLife.23533)
Supplement: Supplementary file 1. — Proteins identified by LC-MS/MS in human spliceosomal B and Bact complexes, as well as complexes stalled in the presence of compound 028 (B028). Total spectral counts of sequenced peptides are shown. Peptides and proteins were identified by searching fragment spectra against the NCBI database (taxonomy human) using Mascot as search engine and were annotated with Scaffold software. Proteins are grouped according to function or association. Common contaminants, such as ribosomal proteins, are not shown. DOI: http://dx.doi.org/10.7554/eLife.23533.035 [file elife-23533-supp1.docx]

**Supplementary File 1. Protein composition of A^028^ and B^028^ complexes as determined by mass spectrometry.** Proteins identified by LC-MS/MS in human spliceosomal complexes B, B^act^ and complexes stalled in the presence of compound 028 (B^028^). Total spectral counts of sequenced peptides are shown. Peptides and proteins were identified by searching fragment spectra against the NCBI database (taxonomy human) using Mascot as search engine and were annotated with Scaffold software. Proteins are grouped according to function or association. Common contaminants, such as ribosomal proteins, are not shown.
